# Evaluating the Impact of Post-Transplant Cyclophosphamide and Anti-Thymocyte Globulin on CMV Reactivation Following Allogeneic Hematopoietic Stem Cell Transplantation: A Systematic Literature Review

**DOI:** 10.3390/jcm12247765

**Published:** 2023-12-18

**Authors:** Jarosław Dybko, Ugo Giordano, Justyna Pilch, Jakub Mizera, Artur Borkowski, Izabela Dereń-Wagemann

**Affiliations:** 1 Lower Silesia Centre for Oncology, Pulmonology and Hematology in Wrocław, 53-439 Wroclaw, Poland; dybko@post.pl (J.D.); izabeladw@gmail.com (I.D.-W.); 2 Department of Oncology and Hematology, Faculty of Medicine, Wroclaw University of Science and Technology, 50-370 Wroclaw, Poland; 3 Department and Clinic of Endocrinology, Diabetes and Isotope Therapy, Wroclaw Medical University, Wybrzeże Pasteura 4, 50-367 Wroclaw, Poland; 4 Faculty of Medicine, Wroclaw Medical University, 50-367 Wroclaw, Poland; justyna.pilch@student.umw.edu.pl (J.P.); jakub.mizera@student.umw.edu.pl (J.M.); 5 Department of Nuclear Medicine and Endocrine Oncology, M. Sklodowska-Curie National Research Institute of Oncology Gliwice Branch, 44-102 Gliwice, Poland; artur.borkowski.md@gmail.com

**Keywords:** anti-thymocyte globulin, post-transplantation cyclophosphamide, allogeneic stem cell transplantation, CMV reactivation

## Abstract

Anti-thymocyte globulin (ATG) and post-transplantation cyclophosphamide (PTCy) are two frequently utilised strategies in graft-versus-host disease (GvHD) prophylaxis following allogeneic hematopoietic cell transplantation (allo-HCT), currently approved for different recipient-donor settings. In addition, being efficacious in preventing GvHD owing to their T-cell depleting capacity, the employment of these two agents increases the risk of infections, including CMV reactivation, which stands as one of the most common and serious infections following allo-HCT. We performed a systematic literature review of articles published until 1 September 2023, through PubMed, MEDLINE, and Scopus, with the main endpoint being CMV reactivation after PTCy or ATG allo-HCT. The majority of the studies included in the analysis provide supporting evidence for a reduced risk of CMV reactivations following the use of PTCy compared to ATG, although not all findings reached statistical significance. Additionally, it appears that utilising a haploidentical donor leads to a higher incidence of CMV infections and clinically significant CMV infections (CS-CMVis) compared to other donor settings in PTCy allo-HCT. This study aims to compare the risk of CMV infections following allo-HCT in patients who have received either ATG or PTCy as GvHD prophylaxis and discuss other factors that could influence the infectious outcomes of patients who have undergone allo-HCT.

## 1. Introduction

Allogeneic hematopoietic stem cell transplantation (allo-HCT) is a potentially curative treatment for patients suffering from haematological malignancies, as it has been found that transplanted donor cells exhibit a graft-versus-leukaemia effect (GvL) [1,2]. Nevertheless, the positive outcomes of allo-HCT are limited by graft-versus-host disease (GvHD), which is a paramount cause of transplant-related mortality (TRM) [3]. HLA-matched related (MRD) and unrelated (MUD) donor settings have been revealed to be more beneficial compared to the use of HLA-mismatched related (MMRD) or unrelated (MMUD) donors [4,5]. Divergences in the frequency of HLA haplotypes across diverse racial or ethnic groups cause substantial variations in the odds of a patient finding a compatible MUD [6]. Within the Euro-Caucasian population, individuals have a 75% chance of finding a MUD, whereas this probability diminishes for racial or ethnic minorities, with a mere range of 15% up to 45% [7,8]. Unfortunately, MRDs are only available for 30% of patients [9]. Hence, to mitigate the occurrence of GvHD, physicians employ a range of prophylactic strategies, such as in vivo T-cell depletion (TCD) through the administration of pretransplant anti-thymocyte globulin (ATG) [10,11], as well as post-transplantation cyclophosphamide (PTCy) [12].

The effectiveness of PTCy is a result of the induction of dysfunction in proliferating alloreactive donor T-cells, stimulation of proliferation of regulatory T-cells, and sparing effect on non-alloreactive T-cells, which are responsible for the anti-tumour immunity and anti-infectious effect [13,14]. Furthermore, the surviving alloreactive T-cells are actively suppressed by Tregs and other regulatory cells [14]. In an HLA-mismatched donor setting, alloreactive donor T-cells recognise major histocompatibility antigens, whereas in an HLA-matched setting, donor T-cells recognise minor antigens, and, as a consequence, they do not proliferate as rapidly in the HLA-matched setting compared to the HLA-mismatched setting [14,15]. Thus, there is an ongoing debate about PTCys effectiveness following HLA-matched allo-HCT.

A wealth of evidence derived from numerous studies has substantiated PTCy, tacrolimus (Tac), and mycophenolate mofetil (MMF) employment to result in reliable engraftment and reduce the incidence of GvHD in both HLA-matched [16,17] and HLA-mismatched donor scenarios, such as haplo-HSCT [18]. Encouraged by these promising outcomes in haplo-HSCT, further research has subsequently expanded PTCy, Tac, and MMF application to encompass other donor types, including MMUD, where conventional GvHD prophylaxis, often including various ATG formulations in conjunction with other immunosuppressive agents, was not sufficient to prevent high rates of GvHD [19,20]. As per the consensus-based recommendations conceived by an international expert panel [21], the administration of ATG is strongly advised as a part of the myeloablative conditioning (MAC) regimen before bone marrow (BM) and peripheral blood stem cell (PBSC) allo-HCT from a MUD or MMUD as GvHD prophylaxis. With limited evidence, the use of ATG is also recommended before PBSC allo-HCT from MRD. In the context of reduced intensity or nonmyeloablative conditioning (RIC/NMA) regimens, where there is an increased risk of relapse, ATG has demonstrated efficacy in preventing both acute GvHD (aGvHD) and chronic GvHD (cGvHD) [21]. Recently, some new recommendations have been published by the European Group for Bone and Marrow Transplantation (EBMT). According to them, ATG is currently recommended in MRD allo-HCT, while in MMUD and MUD, either PTCy or ATG are considered effective in GvHD prophylaxis [14]. PTCy is regarded as the standard of care in haplo allo-HCT, even in MMUD 4/8 to 7/8 transplants, providing low rates of severe aGvHD and cGvHD and non-relapse mortality (NRM) [14]. PTCy, used as a single agent, has been found effective in MRD/MUD bone marrow transplantation (BMT) [17,22]. However, used singularly, it was not superior compared to Tac/methotrexate (MTX) in MRD/MUD myeloablative conditioning (MAC) BMT [23]. In MRD/MUD reduced intensity conditioning (RIC) peripheral blood stem cell transplantation (PBSCT), PTCy as a single agent was not found safe, contrary to PTCy with the addition of Tac and MMF, which are considered the standard GvHD prophylaxis in this setting [16,24,25,26,27,28]. Although PTCy and ATG have been effective in GvHD prophylaxis, there is still no consensus on which protocol may be more beneficial in different donor and graft source settings [29].

The inclusion of PTCy or ATG as part of the GvHD prophylaxis regimen brings a substantial risk of infection owing to their T-cell depleting capacity [30,31,32], with cytomegalovirus (CMV) being the most common cause of viral infections following allo-HCT [33]. Despite the prophylaxis with letermovir for all CMV seropositive recipients and preemptive therapy, the presence of CMV infection continues to be linked to unfavorable outcomes following allo-HCT [34,35,36]. A recent analysis conducted by the Center for International Blood and Marrow Transplant Research (CIBMTR) revealed that both CMV seropositivity and CMV reactivation independently correlated with higher rates of non-relapse mortality (NRM) and lower rates of overall survival (OS) [37]. Furthermore, various smaller-scale studies suggested a connection between CMV infection and reduced risk of relapse, which has not been confirmed in large-scale trials [38,39,40]. As a consequence of CMV infection’s negative influence on allo-HCT outcomes, physicians are striving to find the most effective GvHD prophylaxis strategy, which would concomitantly limit the number of CMV reactivations. Unfortunately, data comparing CMV reactivation following ATG or PTCy administration are scarce.

In this systematic review, we analyse the outcomes of different studies regarding CMV reactivation after allo-HCT, carrying out a comprehensive comparison of the impact of various GvHD prevention measures comprising either PTCy or ATG.

## 2. Materials and Methods

### 2.1. Systematic Literature Review

We performed a systematic literature review utilising PubMed, MEDLINE, and Scopus, searching both separately and together for keyword variants: anti-thymocyte globulin, post-transplantation cyclophosphamide, allogeneic stem cell transplantation, and CMV reactivation. In addition, analysing various studies, reviews, and meta-analyses, we also investigated their reference lists. The search comprised papers published until 1 September 2023. We embraced studies that addressed the effectiveness of PTCy and ATG as GvHD prophylaxis and reported data concerning CMV infections, including overall CMV reactivations, clinically significant CMV infections (CS-CMVi), median time to CMV reactivation, donor and recipient CMV status, CMV prophylaxis regimen, CD34 dose, and HLA matching. We screened the titles and abstracts first, followed by the full text. The exclusion criteria were: non-English, study design, patient population, outcomes, and financial biases. This Systematic Review has not been registered.

### 2.2. Data Presentation, Extraction, and Endpoints

All the available data from the studies reporting on rates, *p* values, and hazard ratios (HRs) with or without 95% confidence intervals (CIs) were extracted, following the endpoints: CD34 dose, HLA matching, CMV prophylaxis, donor/recipient CMV status, overall CMV reactivations, CS-CMVi, and the median time to CMV reactivation. The secondary outcomes were aGvHD grades II–IV, aGvHD grades III–IV, and overall cGvHD. Not all of the endpoints were reported in each study. The data are presented in Table 1, Table 2, Table 3, Table 4, Table 5 and Table 6. Results with *p* values < 0.05 were deemed statistically significant.

## 3. Results

### 3.1. Systematic Literature Review

**Figure 1:**
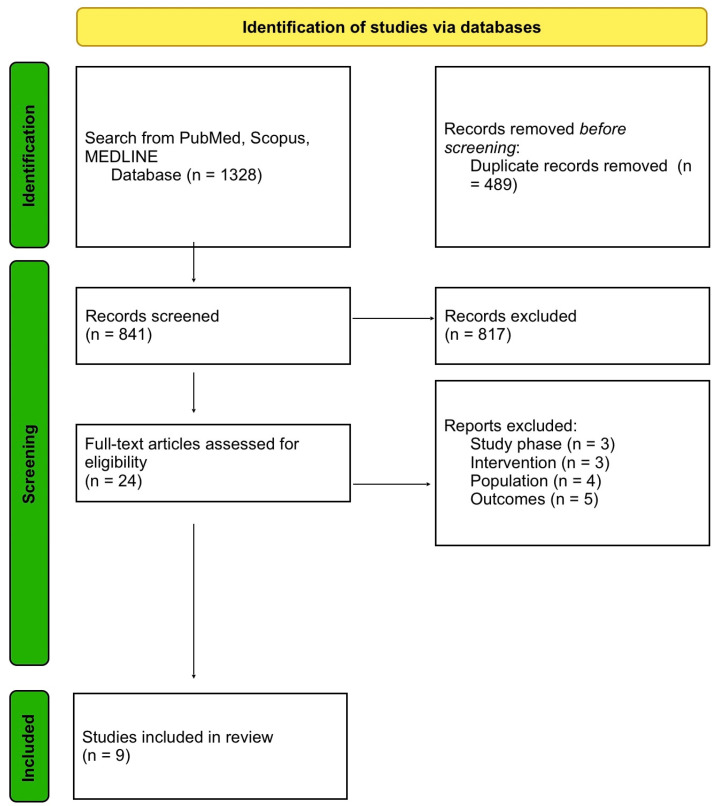
PRISMA flow diagram of this study selection process.

Through meticulous research on PubMed, Scopus, and MEDLINE, we found 1328 citations, which subsequently underwent duplication. The remaining 839 articles were screened based on their titles and abstracts, with 22 studies remaining. After reading the full text of these, nine citations have been included in our paper. They consist of full-text studies published between 2018 and 2023 that analysed the effectiveness of PTCy-based or ATG-based GvHD prophylaxis regimens. The flowchart for the identification of studies is represented in Figure 1.

### 3.2. Outcomes

Most of the studies carried out a direct comparison of PTCy and ATG-based GvHD prophylaxis regimens in different donor settings [26,41,42,43,44,45,46,48], while one included an analysis of three cohorts—ATG MMUD; PTCy MMUD; and haplo PTCy [47]. Among the included trials, considering overall CMV reactivations, a PTCy-based conditioning regimen resulted in fewer CMV infections compared to ATG, with three trials yielding a statistically significant difference (*p* < 0.05) [26,44,46], and three more observing a tendency [41,45,48]. In a direct comparison of ATG MMUD, PTCy MMUD, and PTCy haplo settings [47], there was a remarkable discrepancy in favour of PTCy MMUD in terms of overall CMV reactivations and Cs-CMVi at 100 days compared to ATG MMUD and PTCY haplo (41%, 77%, 63%, *p* = 0.02; 14%, 54%, 53%, *p* = 0.01, respectively). Median time to CMV reactivation was reported in just one article, with PTCy leading to better results compared to ATG in MMUD (39 days vs. 29 days, *p* = 0.02) [41]. The results concerning aGvHD grades II-IV in different donor settings are discrepant, as two studies demonstrated a lower rate when PTCy was used rather than ATG [41,44], and two others suggested a lower rate with ATG [43,45]. Interestingly, a significantly lower occurrence of aGvHD grades III-IV was found in PTCy allo-HCT compared to ATG allo-HCT in mixed donor settings [42,43,44]. As for overall cGvHD, the outcomes are once again contradictory, as two studies showed PTCy to be advantageous [41,42], and in two others, ATG led to better outcomes [43,44].

## 4. Discussion

In the rapidly changing landscape for the treatment of haematological malignancies, including cellular therapies and bispecific antibodies, allogeneic hematopoietic stem cell transplantation (allo-HCT) still remains a valid treatment modality [2]. Nevertheless, GvHD still remains a factor that limits its success [3]. TCD agents such as PTCy and ATG have significantly diminished the incidence of both aGvHD and cGvHD [10,11,12], but they elevate the risk of infections, including CMV reactivations [30,31,32]. Reducing the number of CMV infections following allo-HCT is crucial, as they have been found to increase the rate of NRM and reduce the rate of OS [32]. In this systematic review, we sought to compare the influence of ATG and PTCy on CMV reactivations occurring in patients undergoing allo-HCT in the light of encouraging data and EBMT new standards for PTCy use. According to the current recommendations of the EBMT group for GvHD prevention [14], in MRD, allo-HCT ATG is preferred, with PTCy being a potential therapeutic option. In both MUD and MMUD allo-HCT, the recommendations suggest choosing either ATG or PTCy [14]

In recent years, a number of articles have been published comparing ATG and PTCy in allo-HCT, which included, among other outcomes, reports on CMV infections [26,41,42,43,44,45,46,47,48] Despite not all the results being statistically significant, most of these studies have shown a tendency towards a lower occurrence of overall CMV reactivations and CS-CMVis when PTCy was employed rather than ATG [26,41,42,44,45,46,47,48]. The study by Massoud et al. [43] compared ATG and PTCy in allo-HCT from MUD, MMUD, MRD, and MMRD, reporting no significant differences regarding CMV reactivations between the two cohorts (46% vs. 50%, respectively). Also, it was the only enrolled study with a moderately higher incidence of CMV infections in the PTCy cohort. A similar outcome can be found in the prospective trial by Retière et al. [45], where ATG in allo-HCT from MUD/MRD/MMUD and PTCy in allo-HCT from MUD/MRD/haplo were analysed. Negligible variation in CMV reactivation rates was noted between the two groups (ATG 40% vs. PTCy 27%, *p* = NS); however, the cohorts were small (ATG *n* = 15, PTCy *n* = 30). Among the citations included in our review, a comparison of ATG and PTCy in MMUD allo-HCT has been carried out in two of them [41,42]. Neither Modi et al. [41] nor Jimenez et al. [42] demonstrated statistically significant differences between ATG and PTCy in MMUD regarding CS-CMVis; however, patients administered ATG showed a tendency to develop more CS-CMVis (respectively, 6% vs. 0%, *p* = 0.07; 57% vs. 30%, *p* = 0.1). Interestingly, Modi et al. [41] reported data on the median time to CMV reactivation, which were longer in the case of PTCy than ATG (39 days vs. 29 days, *p* = 0.02). The study by Dybko et al. [44] had two cohorts, which comprised patients treated with ATG in MMUD allo-HCT and PTCy in haplo/MMUD allo-HCT, with the latter group developing substantially fewer CMV infections (46.4% vs. 68.8%, *p* = 0.022). Likewise, Mehta et al. [46] demonstrated the superiority of the PTCy regimen compared to ATG in a MUD setting in regard to CMV reactivations (24% vs. 35%, *p* = 0.002). This results are supported by the outcomes of the study by Moiseev et al. [26], where a prophylaxis based on PTCy rather than ATG resulted in fewer CMV reactivations in a MUD/MMUD allo-HCT setting (46.5% vs. 60%, *p* = 0.045, respectively). In the study by Camargo et al. [47], the 100-day and 200-day cumulative incidence of CMV reactivation for ATG MMUD, PTCy MMUD, and PTCy haplo were as follows: 77%, 41%, 63% (*p* = 0.02), and 86%, 64%, and 68% (*p* = 0.049), respectively. These results are similar with respect to CS-CMVis, with lower rates of CS-CMVi in the PTCy MMUD group compared to PTCy haplo and ATG MMUD (14%, 53%, 54% at day 100 *p* = 0.01, and 25%, 53%, 58% at day 200 *p* = 0.03, respectively). Despite not being statistically significant, the rate of 200-day CS-CMVi was reduced in the PTCy MMUD cohort compared to ATG MMUD, regardless of letermovir treatment (25% vs. 58%, *p* = 0.06). After adjusting for letermovir prophylaxis, the association between a lower risk of CS-CMVi and PTCy MMUD remained significant (odds ratio = 0.23, 95% confidence interval, 0.07–0.81, *p* = 0.02) [47]. A recent review and meta-analysis by Tang et al. [29] discussed the impact of ATG and PTCy in unrelated donor allo-HCT. Considering the RR value, no statistically significant differences were found between the PTCy and ATG groups in CMV reactivations and CS-CMVi (RR = 0.89, 95% CI 0.63–1.24, *p* = 0.07, I2 = 57%). An analysis of the patients reported to the Center for International Blood and Marrow Transplantation Research (CIBMTR) who received haplo PTCy, MRD PTCy, or MRD calcineurin inhibitor-based regimens demonstrated that PTCy carries a significant risk of CMV infection regardless of the donor source, which is more pronounced in seropositive patients [49]. In contrast, a recent literature review performed by Mikulska et al. [33] underlined the importance of haploidentical donors, and not PTCy itself, as a risk factor for developing viral infections (including CMV).

Although the aim of our study was to compare the influence of PTCy and ATG regimens on CMV reactivations, it is important to note that other factors may also impact the infectious outcomes after allo-HCT. For instance, a haploidentical donor setting with PTCy has been found to significantly contribute to the development of CMV infection compared to PTCy MMUD [47]. Furthermore, in patients that have developed a CMV infection, the combination of PTCy and a haploidentical donor is synergistic for lower OS and higher NRM, which is especially pronounced in seropositive recipients [32]. Notably, recipient seropositivity, regardless of PTCy or ATG employment, was independently associated with a higher risk of grade II-IV aGvHD [50]. A yet unpublished report by Little et al. on behalf of the EBMT addressing opportunistic infections in patients receiving PTCy in haplo and URD settings found that haplo PTCy compared to PTCy from MUD/MMUD resulted in a higher rate of CS-CMVi (25% vs. 15%, respectively; *p* = 0.03) [51]. This would suggest a negative impact on CMV-related outcomes in the haplo-donor setting when PTCy is used.

In recent years, there have also been studies that sought to determine the impact of combining ATG and PTCy on GvHD and CMV reactivations [52,53,54], of whom two have employed this novel regimen in a MUD PBSC allo-HCT setting [52,54], and one in haplo allo-HCT [53]. In MUD allo-HCT, ATG + PTCy did not provide any extra benefits compared to ATG alone in terms of better OS, GRFS, or GvHD; also, NRM caused by infections did not differ between the two groups [52]. Nevertheless, a prospective, multi-centre trial carrying out a direct comparison of PTCy and PTCy + ATG in the same setting resulted in a promising outcome. It was demonstrated that the cumulative incidences of both cGvHD and grade II-IV aGvHD were significantly lower in the PTCy + ATG group (24.5% vs. 47.1%, *p* = 0.017; 14.1% vs. 33.3%, *p* = 0.013), with a concomitant improvement of NRM and GRFS (13.2% vs. 34.5%, *p* = 0.049; 67.3% vs. 42.3%, *p* = 0.032) [54]. However, in terms of the 100-day CMV reactivation incidence, the results were comparable between the two cohorts (50.9% vs. 47.1%, *p* = 0.692) [54]. In haplo PBSC allo-HCT, it has been found that low-dose ATG + PTCy may effectively prevent aGvHD with a significantly lower CMV reactivation rate than that with high-dose ATG and a similar incidence of CMV to that of a standard PTCy regimen [53]. A retrospective study on behalf of the EBMT presented insights into the viability of incorporating ATG with PTCy in haplo-PBSCT. The study observed a reduced occurrence of cGVHD and an enhanced, more rapid neutrophil engraftment. However, there were no observed differences in the rates of relapse and NRM, with no data on CMV reactivation [55]. Conversely, in a multicenter retrospective study led by El Cheikh, the efficacy of adding ATG to PTCy was compared with the use of PTCy alone in haplo-PBSCT treatments for various haematological malignancies. The findings indicated that there were no substantial differences in transplantation results between the two treatment strategies [56]. Although there have been encouraging outcomes in haploidentical settings, the addition of ATG to PTCy continues to raise concerns, particularly regarding infection rates observed in certain studies [57]. Consequently, there is an ongoing need for extensive, randomised controlled trials to establish this combination as a new standard for GvHD prophylaxis.

This study also has some limitations. First, neither methodological quality nor the risk of bias assessment were included. Second, besides the study by Retière et al. [45], most of the trials enrolled in our systematic review were of retrospective nature [41,42,43,44,46,47]. Moreover, the doses and types of ATG administered, CMV prophylaxis, donor settings, and distribution of baseline characteristics of patients may have been uneven.

In conclusion, the risk of developing a CMV infection following allo-HCT depends on the instituted conditioning regimen, donor setting, type of CMV prophylaxis, and CMV serological status of both the donor and the recipient. The results of the comparison between PTCy and ATG are discrepant, as some of the included studies corroborate a lower risk of CMV reactivations after PTCy than ATG, although not all results were deemed statistically significant. Conversely, a few citations demonstrated no significant impact of either ATG or PTCy on infectious outcomes after allo-HCT. A haploidentical donor setiting increases the rate of CMV reactivation and CS-CMVis compared to other settings in PTCy allo-HCT [47]. In terms of the rates of aGvHD grades II-IV and overall cGvHD in various donor settings with ATG or PTCy, the outcomes of the included studies are contradictory [26,41,42,43,44,45,46,47,48]. However, some studies demonstrated statistically significant variations in aGvHD grades III-IV occurrence in favour of PTCy rather than ATG allo-HCT in mixed donor settings [26,42,43,44,48]. In a MMUD setting, patients receiving a GvHD prophylaxis based on ATG rather than PTCy tended to develop more CMV reactivations and CS-CMVis [41,42], with one study yielding statistical significance in this aspect [47]. Similar results demonstrating a reduced occurrence of CMV reactivations and CS-CMVis with PTCy compared to ATG were obtained when the majority [26,48] or all of the donors [46] were MUD.

Despite the presented results, there is a need for large-scale, multicenter, randomised controlled trials in order to validate the findings of our literature review.

## Figures and Tables

**Table 1:** Patients characteristics.

	Modi et al., 2021, [41], *n* = 76		Jimenez et al., 2022, [42], *n* = 128		Massoud et al., 2022, [43], *n* = 599		Dybko et al., 2023, [44], *n* = 145		Retière et al., 2018, [45], *n* = 45	
Characteristic	ATG	PTCy	ATG	PTCy	ATG	PTCy	ATG + CsA + Mtx	PTCy + TAK+ MMF	ATG	PTCy
Number of patients	*n* = 51	*n* = 25	*n* = 46	*n* = 82	*n* = 476	*n* = 123	*n* = 35	*n* = 110	*n* = 15	*n* = 30
Age (years) median (range)	53 (22–80)	62 (31–76)	55 (21–72)	60 (21–75)	50 (SD = 14)	50 (SD = 13)	<60 years 91.4% >60 8.6%	<60 years 80.9% >60 19.1%	65 (32–72)	62 (24–72)
Sex, (%) (M)ale (F)emale	M: *n* = 27 (53%) F: *n* = 24 (47%)	M: *n* = 13 (52%) F: *n* = 12 (48%)	M: *n* = 24 (52%) F: *n* = 22 (48%)	M: *n* = 45 (55%) F: *n* = 37 (45%)	M: *n* = 294 (52%) F: *n* = 182 (48%)	M: *n* = 75 (60%) F: *n* = 48 (40%)	M: *n* = 18 (51.4%) F: *n* = 17 (48.6%)	M: *n* = 56 (50.9%) F: *n* = 54 (49.1%)	M: *n* = 8 (53%) F: *n* = 7 (47%)	M: *n* = 23 (77%) F: *n* = 7 (23%)
Follow-up median (range)	5.27 years	1.13 years	45.7 months (3.7–106)	27 months (6.6–58.7)	16 months (1–169)	16 months (1–169)	NR	NR	24 months	24 months
Diagnosis	AML: *n* = 40 (78%) MDS: *n* = 11 (22%)	AML: *n* = 19 (76%) MDS: *n*= 6 (24%)	NR	NR	ALL: *n* = 27 (6%) AML: *n* = 206 (43%) CML: *n* = 16 (3%) MDS: *n* = 43 (9%) MDS-MPN: *n* = 6 (1%) HL: *n* = 4 (1%) NHL: *n* = 75 (16%) MM: *n* = 64 (13%) PMF: *n* = 12 (3%) Other AL: *n* = 3 (1%)	ALL: *n* = 35 (29%) AML: *n* = 23 (19%) CML: *n* = 1 (1%) MDS: *n* = 2 (2%) MDS-MPN: *n* = 4 (3%) HL: *n* = 2 (2%) NHL: *n* = 13 (11%) MM: *n* = 38 (31%) PMF: *n* = 2 (2%) Other AL: *n* = 3 (2%)	AML + MDS *n* = 14 (40%) ALL *n* = 7 (20%) HL + NHL + MM *n* = 11 (31.4%) OMF, CML, SAA *n* = 3 (8.6%)	AML + MDS *n* = 52 (47.3%) ALL *n* = 15 (13.6%) HL + NHL + MM *n* = 31 (28.2%) OMF, CML, SAA *n* = 12 (10.9%)	MDS: *n* = 3 (20%) AML: *n* = 7 (35%) ALL: *n* = 2 (13%) Lymphoma *n* = 0 (0%) Hodgkin disease: *n* = 2 (13%) MF: *n* = 0 (0%) CLL: *n* = 0 (0%) CML: *n* = 1 (7%)	MDS: *n* = 4 (13%) AML: *n* = 11 (37%) ALL: *n* = 2 (7%) Lymphoma *n* = 1 (3%) Hodgkin disease: *n* = 3 (10%) MF: *n* = 7 (23%) CLL: *n* = 1 (3%) CML: *n* = 1 (3%)

Abbreviations: AML—acute myeloid leukaemia; CML—chronic myeloid leukaemia; CMML—chronic myelomonocytic leukaemia; ALL—acute lymphoblastic leukaemia; MPD—myeloproliferative disorders; MDS—myelodysplastic syndrome; MDS-MPN—myelodysplastic syndrome—myeloproliferative neoplasm; HL—Hodgkin lymphoma; NHL—non-Hodgkin lymphoma; MM—multiple myeloma; MF—myelofibrosis; Other AL—other acute leukemia; MUD—matched unrelated donor; MRD—matched related donor; MMRD—mismatched related donor; MMUD—mismatched unrelated donor; CS-CMVi—a clinically significant CMV infection; NR—not reported; SD—standard deviation; NS—not significant.

**Table 2:** Patients characteristics.

	Mehta et al., 2022, [46], *n*= 552		Camargo et al., 2021, [47], *n* = 78			Bailén et al., 2021, [48], *n* = 132		Moiseev et al., 2016, [26], *n* = 211	
Characteristic	ATG + Tac/MTX	PTCy	ATG MMUD	PTCy MMUD	PTCy haplo	ATG + MTX + CsA	PTCy	ATG	PTCy
Number of patients	*n* = 306	*n* = 246	*n* = 37	*n* = 22	*n* = 19	*n* = 60	*n* = 72	*n* = 125	*n* = 86
Age (years) median (range)	29 (18–59)	29 (18–60)	54 (39–63)	60 (50–62)	48 (46–59)	42 (31–55)	44 (18–72)	31 (18–62)	34 (18–59)
Sex, (%) (M)ale (F)emale	NR	NR	M: *n* = 15 (41%) F: *n* = 22 (59%)	M: *n* = 9 (41%) F: *n* = 13 (59%)	M: *n* = 10 (53%) F: *n* = 9 (47%)	M: *n* = 37 (62%) F: *n* = 23 (38%)	M: *n* = 42 (57%) F: *n* = 30 (43%)	NR	NR
Follow-up median (range)	53 (16–79)	29 (3–64)	259 days (98–531)	228 days (155–370)	217 days (148–368)	78 months (12–125)	26 months (6–65)	17 months (1–64)	12 months (4–30)
Diagnosis	AML/MDS *n* = 158 (52%) ALL *n* = 63 (21%) Chronic lymphoid malignancies *n* = 63 (21%) Chronic myeloid malignancies ^1^ *n* = 22 (7%)	AML/MDS *n* = 184 (75%) ALL *n* = 10 (4%) Chronic lymphoid malignancies *n* = 15 (6%) Chronic myeloid malignancies ^1^ *n*= 37 (15%)	AL: *n* = 19 (51%) Lymphoma: *N* = 5 (14%) MDS/MPN: *n* = 10 (27%) Other: *n* = 3 (8%)	AL: *n* = 16 (73%) Lymphoma: *n* = 2 (9%) MDS/MPN: *n* = 3 (14%) Other: *n* = 1 (5%)	AL: *n* = 9 (47%) Lymphoma: *n* = 4 (21%) MDS/MPN: *n* = 2 (11%) Other: *n* = 4 (21%)	AML/MDS: *n* = 35 (58%) ALL: *n* = 13 (22%) NHL/CLL: *n* = 8 (13%) Others *n* = 4 (7%)	AML/MDS: *n* = 47 (65%) ALL: *n* = 18 (25%) NHL/CLL: *n* = 2 (3%) Others *n* = 5 (7%)	AML: *n* = 83 (66%) ALL: *n* = 42 (34%)	AML: *n* = 52 (60%) ALL: *n* = 34 (40%)

Abbreviations: AML—acute myeloid leukaemia; CML—chronic myeloid leukaemia; CMML—chronic myelomonocytic leukaemia; ALL—acute lymphoblastic leukaemia; MPD—myeloproliferative disorders; MDS—myelodysplastic syndrome; MDS-MPN—myelodysplastic syndrome—myeloproliferative neoplasm; HL—Hodgkin lymphoma; NHL—non-Hodgkin lymphoma; MM—multiple myeloma; MF—myelofibrosis; Other AL—other acute leukemia; MUD—matched unrelated donor; MRD—matched related donor; MMRD—mismatched related donor; MMUD—mismatched unrelated donor; CS-CMVi—a clinically significant CMV infection; NR—not reported; SD—standard deviation; NS—not significant. ^1^ Chronic myeloid malignancies include acute myeloid leukaemia and other myeloproliferative disorders.

**Table 3:** Data regarding conditioning regimens, donor type, GVHD-related aspects, and survival.

	Modi et al., 2021, [41], *n* = 76		Jimenez et al., 2022, [42], *n* = 128		Massoud et al., 2022, [43], *n* = 599		Dybko et al., 2023, [44], *n* = 145		Retière et al., 2018, [45], *n* = 45	
Characteristic	ATG	PTCy	ATG	PTCy	ATG	PTCy	ATG + CsA + Mtx	PTCy + TAK+ MMF	ATG	PTCy
Number of patients	*n* = 51	*n* = 25	*n* = 46	*n* = 82	*n* = 476	*n* = 123	*n* = 35	*n* = 110	*n* = 15	*n* = 30
Conditioning	MAC: *n* = 30 RIC: *n* = 21	MAC: *n* = 5 RIC: *n* = 20	Busulfan based *n* = 18 (39%) Fludrabine/Cy/TBI-200 *n* = 5 (11%) Melphalan based *n* = 16 (35%) TBI based *n* = 7 (15%) *p* = 0.1	Busulfan based *n* = 25 (31%) Fludrabine/Cy/TBI-200 *n* = 22 (27%) Melphalan based *n* = 28 (34%) TBI based *n* = 7 (9%) *p* = 0.1	Busulfan based *n* = 256 (54%) TBI based *n* = 130 (27%) Other *n* = 90 (19%) *p* < 0.001	Busulfan based *n* = 29 (24%) TBI based *n* = 55 (45%) Other *n* = 39 (32%) *p* < 0.001	RIC *n* = 2 (5.7%) MAC *n* = 30 (85.7%) NMA *n* = 3 (8.6%)	RIC *n* = 19 (17.3%) MAC *n* = 72 (65.5%) NMA *n* = 19 (17.3%)	RIC: *n* = 15 (100%) Clofarabine-based *n* = 15 (50%) Fludarabine-based *n* = 15 (50%)	RIC: *n* = 30 (100%) Clofarabine-based *n* = 10 (66%) Fludarabine-based *n* = 5 (33%)
Stem cell source, %	Bone marrow: *n* = 2 (4%) Peripheral blood: *n* = 49 (96%)	Bone marrow: *n* = 1 (4%) Peripheral blood: *n* = 24 (96%)	Bone marrow: *n* = 53 (41%) Peripheral blood: *n* = 75 (59%)	Bone marrow: *n* = 41 (50%) Peripheral blood: *n* = 41 (50%)	Peripheral blood: *n* = 476 (100%)	Peripheral blood: *n* = 123 (100%)	Peripheral blood: *n* = 35 (100%)	Peripheral blood: *n* = 110 (100%)	Peripheral blood: *n* = 15 (100%)	Peripheral blood: *n* = 30 (100%)
Donor	MMUD	MMUD	MMUD	MMUD	MRD *n* = 74 (16%) MMRD *n* = 3 (1%) MUD *n* = 303 (64%) MMUD *n* = 96 (20%)	MRD *n* = 31 (25%) MMRD *n* = 14 (11%) MUD *n* = 57 (46%) MMUD *n* = 21 (17%)	haploidentical *n* = 0 (0%) MMUD *n* = 35 (100%)	haploidentical *n* = 93 (84,5%) MMUD *n* = 17 (15.5%)	MUD *n* = 6 (40%) MRD *n* = 8 (53%) MMUD *n* = 1 (7%)	Haploidentical *n* = 20 (66%) MUD *n* = 6 (20%) MRD *n* = 4 (14%)
aGvHD grades II-IV	52.9% (*p* = 0.01)	24.4% (*p* = 0.01)	NR	NR	36% (*p* = 0.005)	40% (*p* = 0.005)	*n* = 11 (31.4%) *p* = 0.005	*n* = 19 (17.2%) *p* = 0.005	*n* = 7 (47%)	*n* = 14 (47%)
aGvHD grades III–IV	19.6% (*p* = 0.38)	12% (*p* = 0.38)	31% (*p* = 0.03)	15% (*p* = 0.03)	15% (*p* = 0.005)	12% (*p* = 0.005)	*n* = 7 (20.0%) *p* = 0.005	5 (4.5%) *p* = 0.005	*n* = 3 (20%)	*n* =3 (10%)
cGvHD overall	49% (*p* = 0.006)	16% (*p* = 0.006)	22% (*p* = 0.03)	9% (*p* = 0.03)	15% (*p* = 0.005)	27% (*p* = 0.005)	*n* = 3 (8.6%) *p* = 0.005	*n* = 20 (18.2%) *p* = 0.005	NR	NR
CD34 dose, median (range)	6.61 (1.2–25.58) *p* = 0.299	7.87 (2.21–20.75) *p* = 0.299	2.38 (0.18–9.0) *p* > 0.9	2.34 (0.08–10.8) *p* > 0.9	11.55 (SD = 64)	7.18 (SD = 2)	NR	NR	6.59 (4.57–10.02) *p* = NS	8 (3.9–22) *p* = NS
HLA matching (%)	7/8 (100%)	7/8 (100%)	<7/8 *n* = 1 (2%) 7/8 *n* = 45 (98%) *p* < 0.001	<7/8 *n* = 25 (30%) 7/8 *n* = 57 (70%) *p* < 0.001	10/10 *n* = 377 (SD = 79) <10/10 *n* = 99 (SD = 21)	10/10 *n* = 88 (SD = 72) <10/10 *n* = 35 (SD = 29)	NR	NR	NR	NR
Overall survival	1 year 57% *p* = 0.136	1 year 70% *p* = 0.136	1 year 0.45 2 years 0.29 *p* < 0.001	1 year 0.75 2 years 0.66 *p* < 0.001	3 years 65% *p* = 0.663	3 years 58% *p* = 0.663	5 years 32.4% *p* = 0.03	5 years 51.1% *p* = 0.03	1 year 73% 2 years 73% *p* = NS	1 year 90% 2 years 79% *p* = NS

Abbreviations: MUD—matched unrelated donor; MRD—matched related donor; MMRD—mismatched related donor; MMUD—mismatched unrelated donor; NR—not reported; SD—standard deviation; NS—not significant.

**Table 4:** Data regarding conditioning regimens, donor type, GVHD-related aspects, and survival.

	Mehta et al., 2022, [46], *n* = 552		Camargo et al., 2021, [47], *n* = 78			Bailén et al., 2021, [48], *n* = 132		Moiseev et al., 2016, [26], *n* = 211	
Characteristic	ATG + Tac/MTX	PTCy	ATG MMUD	PTCy MMUD	PTCy haplo	ATG + MTX + CsA	PTCy	ATG	PTCy
Number of patients	*n* = 306	*n* = 246	*n* = 37	*n* = 22	*n* = 19	*n* = 60	*n* = 72	*n* = 125	*n* = 86
Conditioning	MAC *n* = 196 (64%) RIC *n* = 110 (36%)	MAC *n* = 148 (60%) RIC *n* = 98 (40%)	MAC: *n* = 8 (22%) RIC: *n* = 29 (78%)	MAC: *n* = 3 (14%) RIC: *n* = 19 (86%)	MAC: *n* = 1 (5%) RIC: *n* = 18 (95%)	MAC: *n* = 41 (68%) RIC: *n* = 19 (32%)	MAC: *n* = 45 (63%) RIC: *n* = 27 (37%)	MAC: *n* = 32 (26%) RIC: *n* = 93 (74%)	MAC: *n* = 21 (24%) RIC: *n* = 65 (76%)
Stem cell source, %	Peripheral blood *n* = 195 (64%) Bone marrow *n* = 111 (36%)	Peripheral blood *n* = 190 (77%) Bone marrow *n* = 56 (23%)	Bone marrow: *n* = 10 (27%) Peripheral blood: *n* = 27 (73%)	Bone marrow: *n* = 20 (91%) Peripheral blood: *n* = 2 (9%)	Bone marrow: *n* = 2 (11%) Peripheral blood: *n* = 17 (89%)	Bone marrow: *n* = 5 (8%) Peripheral blood: *n* = 55 (92%)	Bone marrow: *n* = 16 (22%) Peripheral blood: *n* = 56 (78%)	Peripheral blood: *n* = 125 (100%)	Peripheral blood: *n* = 86 (100%)
Donor	MUD	MUD	MMUD	MMUD	haploidentical	MUD *n* = 49 (82%) MMUD *n* = 11 (18%)	MUD *n* = 63 (87%) MMUD *n* = 9 (13%)	MUD *n* = 106 (85%) MMUD *n* = 19 (15%)	MUD *n* = 68 (79%) MMUD *n* = 18 (21%)
aGvHD grades II-IV	180-day 42% *p* = 0.03	180-day 52% *p* = 0.03	*n* = 12 (32%) *p* = 0.39	*n* = 4 (18%) *p* = 0.39	*n* = 7 (37%) *p* = 0.39	day +100: 67% *p* = 0.008	day +100: 46% *p* = 0.008	45% *p* = 0.00003	19% *p* = 0.00003
aGvHD grades III-IV	9% *p* = 0.5	8% *p* = 0.5	*n* = 1 (3%) *p* = 0.14	*n* = 3 (14%) *p* = 0.14	*n* = 0 (0%)	day +100: 34% *p* = 0.003	day +100: 3% *p* = 0.003	27% *p* < 0.0001	4% *p* < 0.0001
cGvHD overall	3-year 19% *p* = 0.5	3-year 18% *p* = 0.5	NR	NR	NR	37% *p* = 0.75	37% *p* = 0.75	65% *p* < 0.0001	16% *p* < 0.0001
CD34 dose, median (range)	NR	NR	6.1 (2.8–8.4) *p* < 0.0001	2.0 (1.5–3.4) *p* < 0.0001	8.9 (6.7–14) *p* < 0.0001	4.7 (4–6) *p* = 0.786	5.2 (3.2–7) *p* = 0.786	5.9 (SD 1.5)	6.0 (SD 1.5)
HLA matching (%)	NR	NR	NR	NR	NR	10/10 *n* = 49 (82%) 8/8 *n* = 0 (0%) 9/10 *n* = 11 (18%)	10/10 *n* = 55 (76%) 8/8 *n* = 8 (11%) 9/10 *n* = 9 (13%)	10/10 *n* = 106 (85%) 8–9/10 *n* = 19 (15%)	10/10 *n* = 68 (79%) 8–9/10 *n* = 18 (21%)
Overall survival	3 years 55% *p* = 0.05	3 years 61% *p* = 0.05	NR	NR	NR	2 years 58% *p* = 0.475	2 years 60% *p* = 0.475	69% *p* = 0.0007	40% *p* = 0.0007

Abbreviations: MUD—matched unrelated donor; MRD—matched related donor; MMRD—mismatched related donor; MMUD—mismatched unrelated donor; NR—not reported; SD—standard deviation; NS—not significant.

**Table 5:** Details on CMV prophylaxis, donor and recipient CMV status, and CMV reactivation.

	Modi et al., 2021, [41], *n* = 76		Jimenez et al., 2022, [42], *n* = 128		Massoud et al., 2022, [43], *n* = 599		Dybko et al., 2023, [44], *n* = 145		Retière et al., 2018, [45], *n* = 45	
Characteristic	ATG	PTCy	ATG	PTCy	ATG	PTCy	ATG + CsA + Mtx	PTCy + TAK+ MMF	ATG	PTCy
Number of patients	*n* = 51	*n* = 25	*n* = 46	*n* = 82	*n* = 476	*n* = 123	*n* = 35	*n* = 110	*n* = 15	*n* = 30
CMV prophylaxis	NR	NR	letermovir	letermovir	acyclovir	acyclovir	NR	NR	NR	NR
(D)onor/(R)ecipient CMV status	D+/R+ *n* = 21 (41%) D+/R− *n* = 9 (18%) D−/R+ *n* = 20 (39%) D−/R− *n* = 1 (2%) *p* > 0.99	D + /R+ *n* = 11 (44%) D + /R− *n* = 4 (16%) D−/R+ *n* = 9 (36%) D−/R− *n* = 1 (4%) *p* > 0.99	R+ *n* = 37 (80%)	R+ *n* = 55 (67%)	D+/R+ *n* = 194 (41%) D+/R− *n* = 68 (14%) D−/R+ *n* = 62 (13%) D−/R− *n* = 151 (32%)	D+/R+ *n* = 58 (47%) D+/R− *n* = 15 (12%) D−/R+ *n* = 9 (7%) D−/R− *n* = 41 (34%)	R+ *n* = 29 (82.9%)	R+ *n* = 90 (81.8%)	NR	NR
CMV reactivation overall	42% *p* = 0.07	20% *p* = 0.07	NR	NR	*n* = 214 (46%)	*n* = 60 (50%)	*n* = 24 (68.8%) *p* = 0.022	*n* = 51 (46.4%) *p* = 0.022	*n* = 6 (40%) *p* = NS	*n* = 8 (27%) *p* = NS
CS-CMVi	*n* = 3 (6%) *p* = 0.07	*n* = 0 *p* = 0.07	57% *p* = 0.1	30% *p* = 0.1	NR	NR	NR	NR	NR	NR
Median time to CMV reactivation (days)	29 days *p* = 0.02	39 days *p* = 0.02	NR	NR	NR	NR	NR	NR	NR	NR

Abbreviations: CS-CMVi—clinically significant CMV infection; NR—not reported; SD—standard deviation; NS—not significant.

**Table 6:** Details on CMV prophylaxis, donor and recipient CMV status, and CMV reactivation.

	Mehta et al., 2022, [46], *n* = 552		Camargo et al., 2021, [47], *n* = 78			Bailén et al., 2021, [48], *n* = 132		Moiseev et al., 2016, [26], *n* = 211	
Characteristic	ATG + Tac/MTX	PTCy	ATG MMUD	PTCy MMUD	PTCy haplo	ATG + MTX + CsA	PTCy	ATG	PTCy
Number of patients	*n* = 306	*n* = 246	*n* = 37	*n* = 22	*n* = 19	*n* = 60	*n* = 72	*n* = 125	*n* = 86
CMV prophylaxis	NR	NR	Acyclovir *n* = 33 (89%) Acyclovir/Letermovir: *n* = 4 (11%) *p* = 0.007	Acyclovir *n* = 12 (55%) Acyclovir/Letermovir *n* = 10 (45%) *p* = 0.007	Acyclovir *n* = 16 (84%) Acyclovir/Letermovir *n* = 3 (16%) *p* = 0.007	Acyclovir	Acyclovir	NR	NR
(D)onor/(R)ecipient CMV status	R+ *n* = 259 (84%) R− *n* = 46 (15%) Missing: *n* = 1	R+ *n* = 172 (70%) R− *n* = 74 (30%) Missing: *n* = 0	D+/R− *n* = 2 (5%) *p* = 0.85 D+/R+ *n* = 19 (51%) *p* = 0.84 D−/R+ *n* = 15 (41%) *p* = 0.87 D−/R− *n* = 1 (3%) *p* = 0.06	D+/R− *n* = 2 (9%) *p* = 0.85 D+/R+ *n* = 10 (45%) *p* = 0.84 D−/R+ *n* = 10 (45%) *p* = 0.87 D−/R− *n* = 0 (0%) *p* = 0.06	D+/R− *n* = 1 (5%) *p* = 0.85 D+/R+ *n* = 8 (42%) *p* = 0.84 D−/R+ *n* = 7 (37%) *p* = 0.87 D−/R− *n* = 3 (16%) *p* = 0.06	D+/R− *n* = 4 (7%) D−/R+ *n* = 32 (53%) No serodiscordance *n* = 24 (40%) *p* = 0.533	D+/R− *n* = 3 (4%) D−/R+ *n* = 37 (52%) No serodiscordance *n* = 32 (44%) *p* = 0.533	NR	NR
CMV reactivation overall	35% *p* = 0.002	24% *p* = 0.002	100-day 77% *p* = 0.02 200-day 86% *p* = 0.049	100-day 41% *p* = 0.02 200-day 64% *p* = 0.049	100-day 63% *p* = 0.02 200-day 68% *p* = 0.049	*n* = 37 (51%) *p* = 0.191	*n* = 24 (40%) *p* = 0.191	*n* = 75 (60%) *p* = 0.045	*n* = 40 (46.5%) *p* = 0.045
CS-CMVi	NR	NR	100-day 54% *p* = 0.01 200-day 58% *p* = 0.03	100-day 14% *p* = 0.01 200-day 25% *p* = 0.03	100-day 53% *p* = 0.01 200-day 53% *p* = 0.03	NR	NR	*n* = 41 (32.8%) *p* = 0.177	*n* = 21 (24.4%) *p* = 0.177
Median time to CMV reactivation (days)	NR	NR	NR	NR	NR	NR	NR	NR	NR

Abbreviations: CS-CMVi—clinically significant CMV infection; NR—not reported; SD—standard deviation; NS—not significant.

## Data Availability

Not applicable.

## Abbreviations

The following abbreviations have been used in this manuscript:

AML	acute myeloid leukaemia
ALL	acute lymphoblastic leukaemia
allo-HCT	allogeneic hematopoietic stem cell transplantation
ATG	anti-thymocyte globulin
BM	bone marrow
BMT	bone marrow transplantation
CIs	confidence intervals
CIBMTR	Center for International Blood and Marrow Transplant Research
CML	chronic myeloid leukaemia
CMV	cytomegalovirus clinically significant CMV infections
CS-CMVis	clinically significant CMV infections
EBMT	European Group for Bone and Marrow Transplantation
GvHD	graft-versus-host disease
aGvHD	acute graft-versus-host disease
cGvHD	chronic graft-versus-host disease
GvL	graft-versus-leukaemia
HL	Hodgkin lymphoma
NHL	non-Hodgkin lymphoma
HRs	hazard ratios
MAC	myeloablative conditioning
MDS	myelodysplastic syndrome
MDS-MPN	myelodysplastic syndrome-myeloproliferative neoplasm
MM	multiple myeloma
MMF	mycophenolate mofetil
MRD	matched related donor
MMRD	mismatched related donor
MUD	matched unrelated donor
MMUD	mismatched unrelated donor
MPNs	myeloproliferative neoplasms
MTX	methotrexate
NMA	nonmyeloablative conditioning
NR	not reported
NRM	non-relapse mortality
NS	not significant
OS	overall survival
PBSC	peripheral blood stem cells
PMF	primary myelofibrosis
PTCy	post-transplantation cyclophosphamide
RIC	reduced-intensity conditioning
SD	standard deviation
Tac	tacrolimus
TCD	T-cell depletion
TRM	transplant-related mortality
